# Supplementary figures and images for: Study on the variable length simple pendulum oscillation based on the relative mode transfer method
Source: PLoS One. 2024 Apr 12;19(4):e0299399. doi: 10.1371/journal.pone.0299399 (PMC11014434; doi:10.1371/journal.pone.0299399)

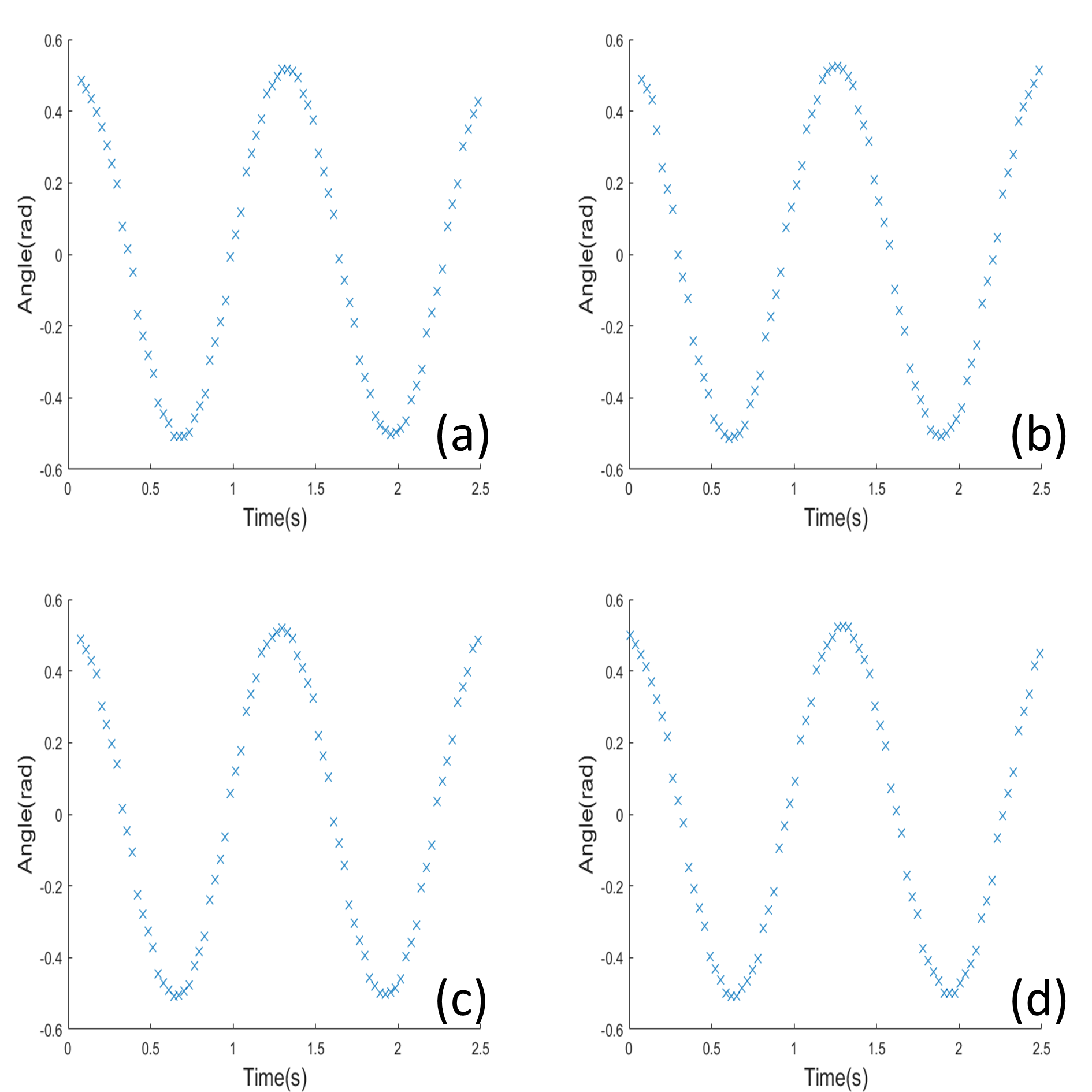

Supplement: S1 Fig — (TIF) [file pone.0299399.s001.TIF]

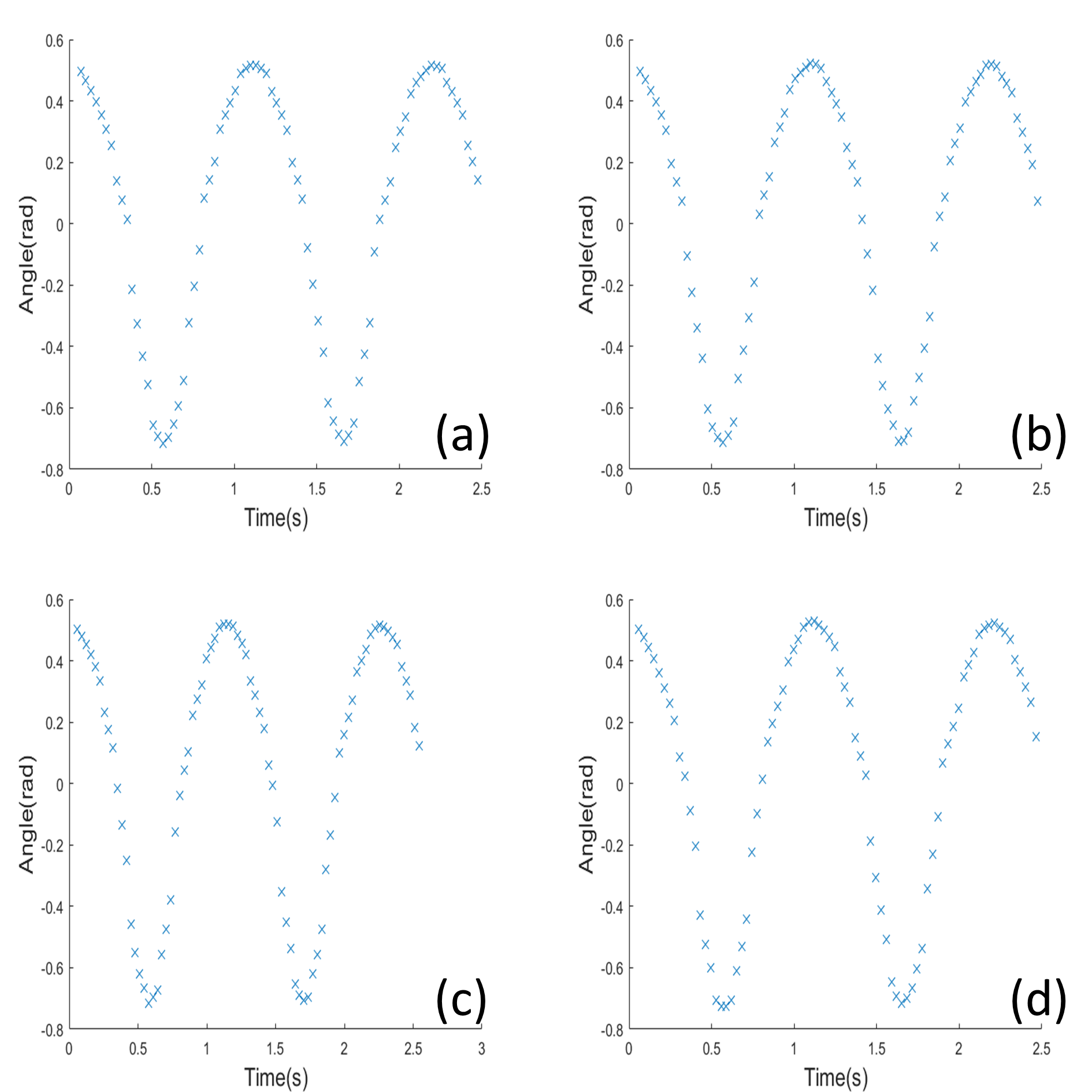

Supplement: S2 Fig — (TIF) [file pone.0299399.s002.TIF]

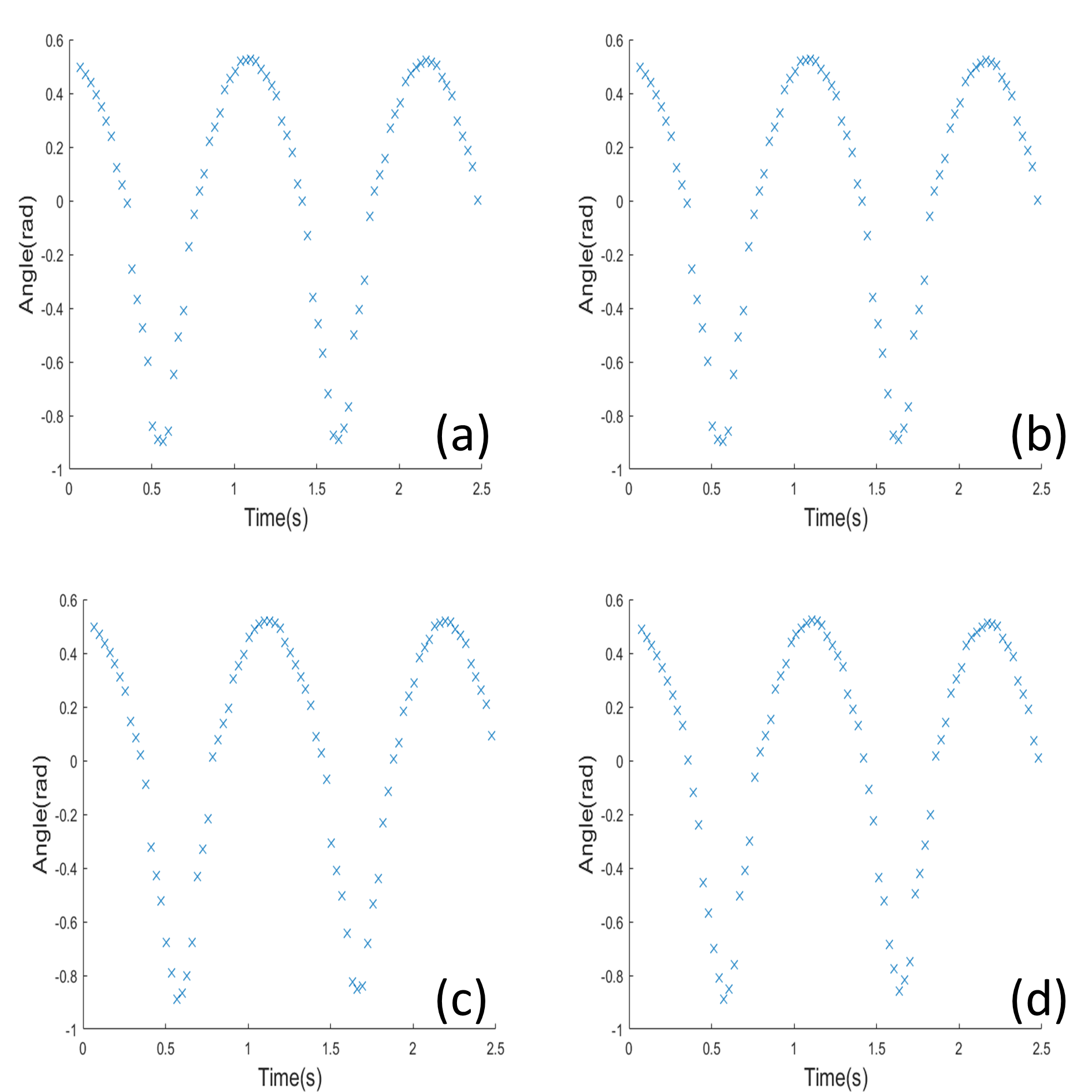

Supplement: S3 Fig — (TIF) [file pone.0299399.s003.TIF]
