# Supplementary material for: Study on the variable length simple pendulum oscillation based on the relative mode transfer method
Source: PLoS One. 2024 Apr 12;19(4):e0299399. doi: 10.1371/journal.pone.0299399 (PMC11014434; doi:10.1371/journal.pone.0299399)
Supplement: Attachment — Submitted filename: Review Plos One.pdf [file pone.0299399.s004.pdf]

I consider the paper has a good level and may be accepted after some improvements.

As a grammar correction I would recommend, in the Introduction, you replace “Wright[11] consider” by “Wright[11] considered…”

In the Eq.(1) you could demonstrate a explanation about this equation. What is the relationship between the damping coefficient ξ and the viscous friction?

After the Eq.(3) you could explain the difference between $l$ and $\bar{l}$.

In Eq.(5) you may avoid misunderstanding if you change the order of the terms. I suggest you write as below. Otherwise, someone may think that $\theta(t_1)$ would be included in the sum.

$$\theta(\mathrm{t}) = \theta(\mathrm{t_i}) + \sum_{j=1}^{i-1} \theta(t_j)$$

As a consequence of changes in Eq.(5), I would recommend to change the same in Eq.(6).

Afterwards, the paper may be published.
